# Traumatic peroneal nerve injury in an adolescent with asymptomatic tibial osteochondroma

**DOI:** 10.11604/pamj.2014.17.145.3997

**Published:** 2014-02-28

**Authors:** Ali Akhaddar, Mustapha Boussouga

**Affiliations:** 1 Department of Neurosurgery, Avicenne Military Hospital, Marrakech, Morocco; 2 University of Mohammed V Souissi, Rabat, Morocco; 3 Department of Orthopedic Surgery, Mohammed V Military Teaching Hospital, Rabat, Morocco

**Keywords:** Nerve injury, osteochondroma, tibia, adolescent

## Image in medicine

A 15-year-old adolescent, previously healthy, presented with right peroneal nerve palsy sustained after a direct closed knee injury in a soccer game. The physical examination revealed a marked weakness to dorsiflexion of the right ankle and toes without sensory loss. A bony mass was noted in the right postero-lateral area of the knee. Plain radiographs and a computed tomography-scan of the knee showed a large osteophytic mass in the proximal metaphyse of the right tibia with an important mass effect on the fibular head which is deformed (A-D). Electrophysiological studies confirmed denervation of the muscles supplied by the right peroneal nerve. The patient underwent surgical decompression of the nerve after resection of the osseous mass. Postoperatively, there was a progressive improvement of the nerve function. The patient was referred for physiotherapy care. Histological features were consistent with benign osteochodroma. Osteochondromas (exostosis) frequently develop around the knee in the distal femur and the proximal tibia and fibula. These osteocartilaginous tumors are usually asymptomatic, but can occasionally impinge on the surrounding nerves and vessels and cause various clinical manifestations. Tibial osteochondroma compressing both the fibular head and the peroneal nerve is rarely seen especially following a direct injury.

**Figure 1:**
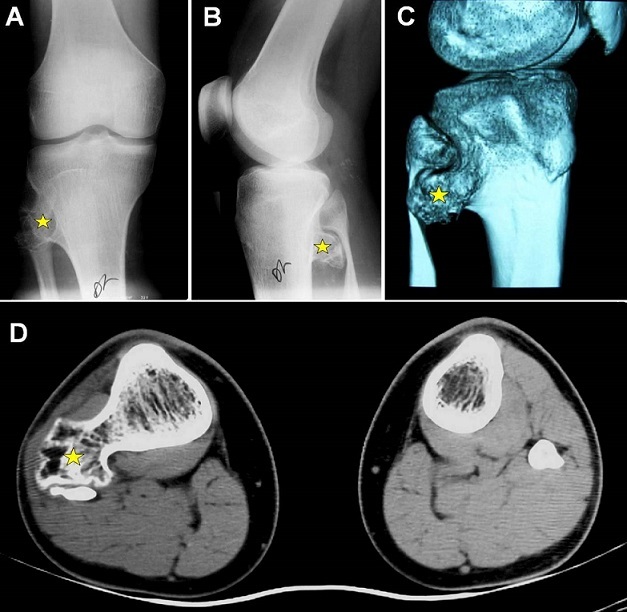
Right knee: Antero-posterior (A) and lateral (B) plain radiographs. 3-D CT-scan bony reconstruction (C) with axial CT-scan of both proximal tibias and fibulas (C-D) showing a large osteophytic bony lesion (star) in the proximal metaphyse of the right tibia with an important mass effect on the fibular head which is deformed (A-D)

